# Transdiagnostic dimensions of psychopathology in chronic tinnitus patients with and without hearing loss

**DOI:** 10.1038/s41598-025-32526-5

**Published:** 2026-01-16

**Authors:** Benjamin Boecking, Kurt Steinmetzger, Petra Brueggemann, Matthias Rose, Birgit Mazurek

**Affiliations:** 1 Tinnitus Center, Charité – Universitatsmedizin Berlin, Charitéplatz 1, 10117 Berlin, Germany; 2 Department of Psychosomatic Medicine, Charité – Universitatsmedizin Berlin, Berlin, Germany

**Keywords:** Tinnitus, Internalizing psychopathology, Transdiagnostic model, Hearing loss, HiTOP, Comorbidity, Diseases, Medical research, Psychology, Psychology, Risk factors

## Abstract

Chronic tinnitus is frequently described as occurring with psychiatric comorbidities. However, this diagnosis-based framing may obscure underlying dimensions of psychological vulnerability in individuals both with and without measurable hearing loss (HL). This study thus aimed to identify transdiagnostic dimensions of psychopathology in patients with chronic tinnitus and examine their relevance for tinnitus-related distress (TRD) in patients with or without HL. In a clinical sample of *N* = 678 chronic tinnitus patients, pure-tone audiometry determined HL status and self-report screeners assessed psychiatric symptom profiles. Multiple regression models tested diagnostic predictors of TRD and diagnosis × HL interactions. Tetrachoric correlations among binary ‘diagnosis pointers’ were subjected to principal axis factor analysis to derive latent dimensions, which were then conceptually linked to the Hierarchical Taxonomy of Psychopathology (HiTOP). Most patients (96%) endorsed at least one diagnosis pointer (median = 5); over half (52.1%) had no identifiable HL. Patients without HL exhibited higher rates of anxiety and substance-related symptoms. TRD was significantly predicted by diagnosis pointers for major depression, agoraphobia, health anxiety, anorexia nervosa, and psychosis. Factor analysis identified three dimensions: Internalizing psychopathology, harmful substance use, and fear-related (social) perceptions. Internalizing psychopathology predicted TRD. HL status was associated with harmful substance use but not with psychological predictors of distress. Findings suggest that chronic tinnitus indexes broad psychological vulnerability rather than discrete psychiatric comorbidities. Whilst inner ear damage may trigger tinnitus onset in patients with HL, persistence across patients—particularly those without HL—suggests a psychological substrate underlying symptom chronicity, distress, and diagnostic covariation. Clinically, the findings support a dimensional, formulation-based approach that situates tinnitus within patients’ broader emotional functioning and foregrounds transdiagnostic processes over discrete diagnoses.

## Introduction

Chronic tinnitus, defined as “*the conscious awareness of a tonal or composite noise for which there is no identifiable corresponding external acoustic source*”^1^, affects a substantial proportion of the population.

While initial symptom onset is frequently associated with peripheral auditory dysfunction, particularly hearing loss (HL^2^), chronicity—and by extension, clinically relevant tinnitus-related distress (TRD)—is largely independent of audiological factors^3^. Instead, a large body of research suggests that emotional and cognitive appraisal processes play a central role in determining whether the tinnitus percept becomes distressing or persistent^3–7^.

These appraisal tendencies reflect broader affective–cognitive processing styles, which are shaped by dispositional and developmental vulnerabilities such as temperament^8,9^, exposure to adverse life events^8–11^, and suboptimal parenting practices or early attachment experiences^8,9,12,13^. In the present paper, we conceptualize these influences from general psychopathology as constituting psychological vulnerability—an umbrella term for enduring affective–cognitive dispositions (e.g. internalizing tendencies, maladaptive appraisal styles, and difficulties in emotion regulation) that increase the likelihood that neutral or ambiguous stimuli, such as a tinnitus percept, are interpreted as distressing.

In individuals with HL, psychological vulnerability may exacerbate distress related to both the tinnitus symptom and the auditory impairment itself. In those without HL, it may directly contribute to the onset and maintenance of tinnitus by fostering a maladaptive interpretation of spontaneously occurring, benign auditory percepts^14,15^. In both cases, the persistence of symptom perception is likely mediated by central rather than peripheral mechanisms, best understood as neuropsychological correlates of subjectively meaningful anxiety and distress^1,16,17^.

The psychiatric literature typically frames the persistent—and at times severe—distress associated with the tinnitus symptom as *comorbidity*, that is, as co-occurring ‘conditions’ such as major depressive, anxiety, and somatoform disorders^18–21^. In some cases, these psychiatrically labelled conditions precede the onset of tinnitus, supporting the role of psychological vulnerability in shaping individuals’ initial appraisal and subsequent experience of the symptom^22,23^.

Traditionally, such comorbidities are classified according to categorical nosological systems such as the International Statistical Classification of Diseases and Related Health Problems (ICD^24^) and Diagnostic and Statistical Manual of Mental Disorders (DSM^25^). However, these systems have been criticized for limited reliability and validity as well as for imposing rigid diagnostic boundaries^26–29^. In response, transdiagnostic and dimensional models of psychopathology have gained considerable scientific traction as evidence-based alternatives^30–38^. Among these, the Hierarchical Taxonomy of Psychopathology (HiTOP) offers a data-driven, dimensional approach that aims to hierarchically organize psychopathology across multiple levels—from broad factors (‘spectra’) via subfactors to specific signs, symptoms and traits—offering an alternative to traditional disorder-based classification systems^32^. Within this framework, many symptoms associated with tinnitus-related distress—particularly anxiety and depressive symptoms^39–42^—are conceptualized as manifestations of a broader **internalizing spectrum**. This dimension of psychopathology, described as involving “problems within the self”^43^, has been linked to transdiagnostic features such as emotion regulation difficulties^44,45^ which may also play a central role in understanding people with chronic tinnitus^46,47^. Moreover, HiTOP also delineates a separate **somatoform spectrum** for disorders characterized by distressing physical symptoms and health preoccupations which may include chronic tinnitus^15^. Notably, internalizing and somatoform problems tend to covary so much so that HiTOP groups them together under an overarching **emotional dysfunction superspectrum**^48^*.*

Despite an intriguing conceptual fit, no study to date has examined psychiatric comorbidity covariation in chronic tinnitus patients to identify latent transdiagnostic dimensions of psychopathology or align them with ideas consistent with the HiTOP. Moreover, few studies have directly compared psychological profiles between chronic tinnitus patients with and without HL. Given that both patient groups experience distress, but only one has identifiable auditory deficits, it is plausible that patients without HL may exhibit even greater psychological vulnerability—though empirical evidence for this hypothesis remains sparse^49–52^. To address these questions, the present study pursued three aims: (1) to compare self-rated psychiatric symptom profiles in chronic tinnitus patients with and without HL; (2) to examine associations of these diagnosis patterns with TRD; and (3) to identify transdiagnostic dimensions of psychopathology based on observed diagnostic covariation, and, if applicable, conceptually associate them with the HiTOP framework.

## Method

### Participants

The present study reports data from *N* = 678^1^ patients with chronic tinnitus (symptom duration > 3 months) who were seen at the Tinnitus Center, Charité – Universitatsmedizin Berlin, as part of routine clinical care between January 2019 and December 2020.

Patients were eligible for inclusion if they (1) were aged 18 years or older, (2) reported chronic tinnitus (lasting longer than 3 months), (3) had undergone pure-tone audiometry (PTA), (4) had completed a standardized questionnaire battery on an electronic tablet, and (5) had provided written informed consent for anonymized use of their assessment data for research purposes. Exclusion criteria were acute psychotic illness, current addiction, and insufficient German language proficiency.

The sample was gender-balanced (51% female) with participants’ ages ranging from 19 to 82 years (*M*_age_ = 51.7 years; *SD* = 12.2). Ethical approval for data collection and analysis was granted by the Charité ethics committee (No. EA4/216/20).

### Pure tone audiometry

Pure tone audiommetry (PTA) data were extracted from clinical audiometric records, covering frequencies from 0.25 to 8 kHz in 5 dB increments for each ear. We classified HL by (1) computing an average PTA4 hearing threshold (dB) across 0.5, 1, 2, and 4 kHz per ear, (2) recoding the hearing threshold as “normal” (< 20dB), “mild” (20-34dB), “moderate” (35-49dB), “moderate-severe” (50-64dB), “severe” (65-79dB), and “profound” HL (80-94dB) according to hearing loss classification guidelines from the Global Burden of Disease (GBD) expert group^53^, and (3) defining HL as showing at least “mild” impairment (≥ 20dB) in at least one ear^54^. For the right ear, 60.6% had normal hearing (*n* = 411), 27.7% mild (*n* = 188), 8.6% moderate (*n* = 58), 2.5% moderate-to-severe (*n* = 17), 0.3% severe hearing loss (*n* = 2), and 0.3% profound hearing loss (*n* = 2). For the left ear, 59.3% had normal hearing (*n* = 402), 29.8% mild (*n* = 202), 7.1% moderate (*n* = 48), 2.4% moderate-to-severe (*n* = 16), 1.0% severe hearing loss (*n* = 7), and 0.4% profound hearing loss (*n* = 3).

Descriptive analyses showed that 52.1% of patients had no identifiable HL. Compared to those with HL, patients without HL were generally younger and more likely to report more recent tinnitus onset, higher education, single or non-married relationship status, and higher employment rates. See Table 1 for an overview of sample characteristics.

**Table 1:** Sociodemographic information for chronic tinnitus patients with or without hearing loss.

Variable	Total		HL		No HL		
	(*N* = 678)		(*n* = 325)		(*n* = 353)		
	*M*	*SD*	*M*	*SD*	*M*	*SD*	
Age	51.7	12.2	**57.2**	**9.5**	46.6	12.3	*p* <.001
	***n***	***%***	***n***	***%***_***HL***_	***n***	***%***_***noHL***_	
Gender							
Male	332	49.0	165	50.9	167	47.3	-
Female	345	50.9	159	49.1	186	52.7	-
Duration of tinnitus							
*<*1/2 year	83	12.2	36	11.1	47	13.3	-
1/2–1 year	148	21.8	47	14.5	**101**	**28.6**	*p* <.001
1–2 years	96	14.2	36	11.1	**60**	**17.0**	*p* <.05
2–3 years	45	6.6	19	5.9	26	7.4	-
3–4 years	35	5.2	15	4.6	20	5.7	-
*>*4 years	270	39.8	**171**	**52.8**	99	28.0	*p* <.05
Education							
None	6	0.9	5	1.5	1	0.3	-
Primary school	9	1.3	7	2.2	2	0.3	-
General school	41	6.0	**27**	**8.3**	14	4.0	*p* <.05
O-levels	116	17.1	**72**	**22.2**	44	12.5	*p* <.05
A-levels	61	9.0	20	6.2	**41**	**11.6**	*p* <.05
Apprenticeship	142	20.9	75	23.1	67	19.0	-
Polytechnic degree	74	10.9	33	10.2	41	11.6	-
University degree	228	33.6	85	26.2	**143**	**40.5**	*p* <.01
Nationality							
German	619	91.3	302	93.2	317	89.8	-
Other	58	8.6	22	6.8	36	10.2	-
Relationship status							
Single	162	23.9	48	14.8	**114**	**32.3**	*p* <.001
In partnership	69	10.1	22	6.8	**47**	**13.3**	*p* <.01
Married	330	48.7	**183**	**56.5**	147	41.6	*p* <.001
Separated	14	2.1	7	2.2	7	2.0	-
Divorced	85	12.5	**51**	**15.7**	34	9.6	*p* <.05
Widowed	17	2.5	**13**	**4.0**	4	1.1	*p* <.05
Employment status							
Employed	478	70.5	209	64.5	**269**	**76.2**	*p* <.01
Unemployed	199	29.4	**115**	**35.5**	84	23.8	*p* <.01

*Notes.* Comparisons were made using a Univariate Analysis of Variance (age) and Pearson’s *χ*^2^ and post-hoc *Z*-Tests for independent proportions (nominal variables). Boldened numbers illustrate higher frequency (proportions) in the respective patient subgroup. *M* = mean, *SD* = standard deviation, HL = hearing loss.

^†^GBD expert group classification^53^.

### Questionnaire measures

For the purposes of the present analyses, we derived a set of twelve binary “diagnosis pointers” that indexed the probable presence versus absence of specific psychiatric syndromes via two self-report questionnaires: the ICD-10 Symptom Rating (ISR) and the Structured Clinical Interview-I (SCID-I) Screening Questionnaire.

#### ICD-10 symptom rating

The ICD-10 Symptom Rating ISR^55–57^, consists of 29 items that are answered on a 5-point-scale (0 = *strongly disagree*, 1 = *disagree somewhat*, 2 = *somewhat agree*, 3 = *agree*, 4 = *strongly agree*).

Subscale scores were dichotomized using the established clinical cut-offs that are validated against ICD-10 diagnoses^58^ such that scores at or above the cut-off indicated a positive screening for (1) Major Depressive Disorder, (2) Agoraphobia with or without Panic Disorder, (3) Obsessive-Compulsive Disorder (cut-off scores: ≥ 1.00), (4) Health Anxiety (cut-off score: ≥ 0.75), and (5) Anorexia Nervosa (cut-off score: ≥ 0.67).

The ISR has demonstrated a stable five-factor structure corresponding to ICD-10 syndrome groups, good internal consistencies, and satisfactory convergent validity in large psychosomatic inpatient and outpatient samples^55,59^. Follow-up work further showed good to excellent retest reliability and medium to large sensitivity to change across diagnostic groups^56^. For the main analyses, each subscale was dichotomized at its established cut-off (0 = below cut-off, 1 = positive screen), yielding binary diagnosis indicators (“diagnosis pointers”) that could be analyzed alongside the dichotomous SCID-I screening items.

#### Structured clinical interview-I (SCID-I) screening questionnaire

The SCID-I Screening Questionnaire is a brief self-report screener derived from the Structured Clinical Interview for DSM-IV Axis I disorders and has been shown to provide an efficient identification of probable Axis I diagnoses in routine clinical settings^60^. In the present study, *yes/no* items probed for (6) Social Anxiety Disorder, (7) Specific Phobia, (8) Bulimia Nervosa, (9) Alcohol Dependency, (10) Drug Dependency, (11) Mania/Bipolar Disorder, and (12) Psychosis.

For the purposes of the present analyses, we use the term “diagnosis pointers” to denote binary (yes/no) self-report indicators of probable diagnostic status (ISR subscale scores above the recommended cut-offs or SCID-I screening items endorsed with “yes”). Harmonizing ISR and SCID data in this way allowed us to model the covariation of syndrome-level indicators across instruments within a single factor analytic framework. Importantly, positive screenings were *not* validated via structured diagnostic interviews, and should thus be interpreted as indicators of probable, rather than confirmed, diagnoses.

#### Tinnitus handicap inventory (THI)

Tinnitus-related distress, i.e. *subjective tinnitus handicap* severity was measured by the German version^61^ of the Tinnitus Handicap Inventory [THI, 62]. The THI is a widely used measure with well-established psychometric properties, including high internal consistency and good test–retest reliability^62^. The scale consists of 25 items that are answered on a 3-point scale (0 = *no*; 2 = *sometimes*; 4 = *yes*) resulting in a total score between 0 and 100. The German version used here has shown comparable internal consistencies and strong convergent validity with other tinnitus measures^61,63^. In the current sample, Cronbach’s α was excellent (0.93).

### Data analysis

First, descriptive analyses summarized the prevalence of each diagnosis pointer (frequencies and percentages) in the total sample and subsamples defined by HL status. Pearson’s *Chi-square* tests and post-hoc *Z*-tests for independent proportions then tested group differences in these prevalences between patients with and without HL.

Second, a univariate ANCOVA tested the main effect of HL status on TRD as measured by the THI total score, controlling for patients’ age, duration of tinnitus, education, relationship-, and employment status.

Third, a series of multiple linear regressions assessed associations between diagnosis pointers and TRD. In these models, the THI total score was specified as dependent variable and the set of 12 diagnosis pointers as independent variables. We (1) examined associations in the total sample and (2) added diagnosis × HL-status interaction terms to test whether these associations differed between patients with and without HL. All models adjusted for age, duration of tinnitus, education, relationship status, and employment status (cf. Table 1). Significant interactions were followed up using simple slope analyses.

Fourth, to explore the covariation structure among the diagnosis pointers, we estimated a tetrachoric correlation matrix and subjected it to a principal axis factor analysis with quartimax rotation. Quartimax rotation was chosen to minimize the number of factors required to explain and interpret the observed variables^64^. Tetrachoric correlations were used because diagnosis pointers are binary indicators assumed to reflect underlying continuous latent traits, justifying their “as-if ordinal” treatment for dimensional reduction. Sampling adequacy and factorability were evaluated using the Kaiser–Meyer–Olkin (KMO) measure and Bartlett’s test of sphericity. The number of factors to retain was determined based on the scree plot and interpretability of the factor solution. In keeping with our transdiagnostic perspective, we interpret the covariation patterns of these diagnosis pointers as observable indicators of broader psychological vulnerability, rather than as overlapping, discrete disorder entities.

Finally, linear and binary logistic regression analyses examined whether the emerging transdiagnostic dimensions predicted TRD or HL status, respectively. Here, factor scores were computed as sum scores of the binary diagnosis pointers that loaded most strongly on each factor in the rotated solution. To preserve the conceptual distinctiveness of each dimension, diagnosis pointers were assigned exclusively to the factor on which they loaded highest.

All analyses were conducted using IBM SPSS Statistics for Windows, Version 29.0 (IBM Corp., Armonk, NY, USA; available at https://www.ibm.com/products/spss-statistics), except for the tetrachoric correlation estimation, which was performed in R (Version 4.3.1^65^) using the *psych* package (Version 2.5.6^66^). For inferential analyses (ANCOVA, linear and logistic regressions, and factor analysis), cases with missing values on any variable included in a given model were excluded using listwise deletion (SPSS default).

## Results

### Diagnostic self-ratings (diagnosis pointers)

**Fig. 1:**
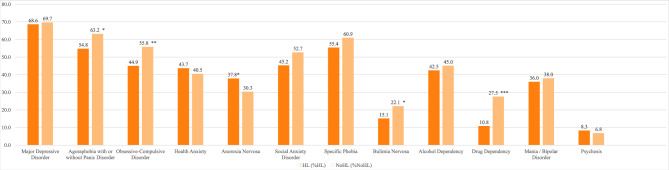
Frequencies of diagnosis pointers in chronic tinnitus patients with or without HL. *Notes*. Percentages in the HL and no HL columns refer to the proportion of patients within each respective subgroup, not the full sample; HL = hearing loss; * = *p* <.05; ** = *p* <.01; *** = *p* <.001.

Of the *N* = 678 patients with chronic tinnitus, *n* = 650 (95.9%) endorsed at least one diagnosis pointer (*range*: 0–12; *Mdn*: 5; *mode*: 5). The most common self-ratings comprised major depressive disorder (69.2% [of the total sample]), followed by agoraphobia with or without panic disorder (59.1%), specific phobia (58.3%), obsessive-compulsive disorder (50.6%), social anxiety disorder (49.1%), alcohol dependency (43.8%), health anxiety (42.0%), mania/bipolar disorder (37.0%), anorexia nervosa (33.9%), drug dependency (19.5%), bulimia nervosa (18.7%), and psychosis (7.5%). Figure 1 illustrates and contrasts frequencies of diagnosis pointers in individuals with or without HL.

### Tinnitus-related distress

Overall, patients reported ‘moderate’ levels of TRD as measured by the THI total score (*M* = 45.54, *SD* = 22.26). Compared to patients without HL, patients with HL reported slightly higher levels of TRD controlling for age, duration of tinnitus, education, relationship-, and employment status (*M*_*HL*_ = 47.91; *SD*_*HL*_ = 23.35;* M*_*NoHL*_ = 43.23; *SD*_*NoHL*_ = 21.01; *F*[1,670] = 6.73, *p* <.05, *partial η*^*2*^ =.01).

To examine associations between diagnosis pointers and TRD across the whole sample, a multiple linear regression analysis specified TRD as dependent variable and the binary diagnosis pointers as independent variables. The overall model had substantial explanatory power (adjusted *R*^*2*^ = 0.39) and revealed that TRD was significantly predicted by diagnosis pointers indexing major depressive disorder, agoraphobia with or without panic disorder, health anxiety, anorexia nervosa, and psychosis (see Table 2).

**Table 2:** Associations between diagnosis pointers and tinnitus-related distress in chronic tinnitus patients.

	*β*	*T*	*p*	*95% CI LL*	*95% CI UL*
Major depressive disorder	0.32	9.32	<.001	12.15	18.65
Agoraphobia with or without panic disorder	0.17	4.65	<.001	4.42	10.88
Obsessive-compulsive disorder	0.07	1.87		−0.15	6.00
Health anxiety	0.24	7.36	<.001	8.05	13.90
Anorexia nervosa	0.08	2.37	<.05	0.60	6.48
Social anxiety disorder	0.03	0.87		−1.59	4.10
Specific phobia	0.01	0.23		−2.58	3.27
Bulimia nervosa	0.04	1.19		−1.46	5.97
Alcohol dependency	0.00	−0.13		−3.03	2.66
Drug dependency	−0.05	−1.68		−6.55	0.51
Mania/bipolar disorder	−0.02	−0.71		−3.82	1.79
Psychosis	0.08	2.49	<.05	1.40	11.83

*Notes.* Diagnosis pointers were included simultaneously in the model using the ENTER approach with the THI_total score specified as dependent variable. CI = confidence interval; LL = lower limit; UL = upper limit.

Comparing the association between diagnosis pointers and TRD in patients with vs. without HL (controlling for patients’ age, duration of tinnitus, education, relationship-, and employment status), only the HL × Agoraphobia (with or without Panic Disorder) interaction emerged as significant (*β* = 0.36, *t* = 7.14, *p* <.05). Simple slope post-hoc analyses revealed that the association between agoraphobia and TRD was significant in both subgroups, but stronger for individuals with compared to those without HL (*β*_*HL*_ = 0.47; *t* = 9.86; *p* <.001, 95% CI [17.71, 26.54], adjusted *R*^*2*^ = 0.28; *β*_*NoHL*_ = 0.36; *t* = 7.17; *p* <.001, 95% CI [32.33, 59.14], adjusted *R*^*2*^ = 0.15).

### Factor analysis

**Fig. 2:**
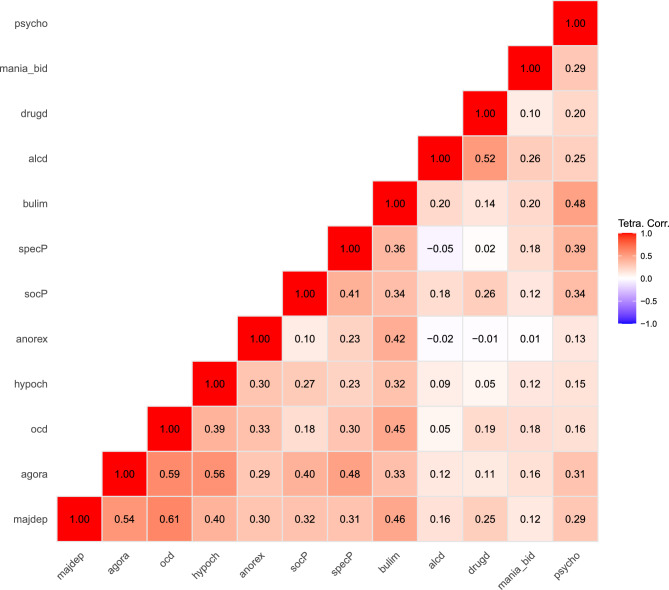
Tetrachoric correlation matrix for the obtained diagnosis pointers. *Notes*. majdep = Major Depressive Disorder; agora = Agoraphobia with or without Panic Disorder; ocd = Obsessive-Compulsive Disorder; hypoch = Health Anxiety; anorex = Anorexia Nervosa; socP = Social Anxiety Disorder; specP = Specific Phobia; bulim = Bulimia Nervosa; alcd = Alcohol Dependency; drugd = Drug Dependency; mania_bid = Mania/Bipolar Disorder; psycho = Psychosis.

Figure 2 illustrates the tetrachoric correlation coefficients for the diagnosis pointers. To identify latent psychological dimensions underlying the covariation among the diagnosis pointers, a principal axis factor analysis with quartimax rotation examined the twelve binary diagnosis variables. Sampling adequacy was confirmed by the KMO measure, which yielded a value of 0.75—considered ‘middling’ to ‘good’ according to Kaiser and Rice^67^. Additionally, Bartlett’s test of sphericity was significant, *χ*^*2*^(66) = 2569.32, *p* <.001, indicating that the correlation matrix was suitable for exploratory factor analysis.

**Fig. 3:**
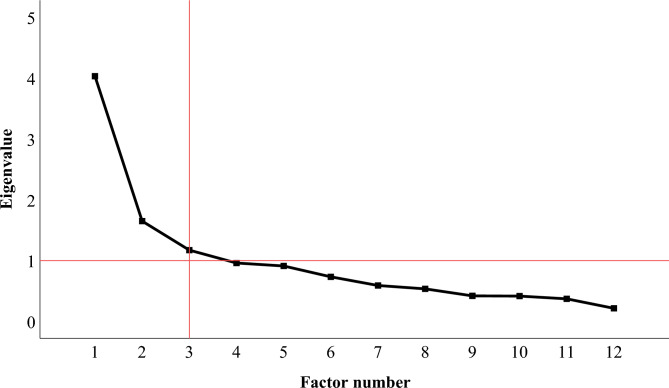
Scree plot for the factor analysis of diagnosis pointers.

The scree plot showed inflexions that justified retaining two or three factors with 47.33% or 57.09% of variance explained, respectively (see Figure 3). To maximize interpretability, account for a broader spectrum of variance, and obtain a more differentiated structure potentially aligning with hierarchical models of psychopathology, we opted for the three-factor solution.

Table 3 lists items and factor loadings following *quartimax*-rotation. Factor 1 was labelled “**internalizing psychopathology**”. It included major depressive disorder, agoraphobia with or without panic disorder, obsessive-compulsive disorder, health anxiety, anorexia nervosa, and bulimia nervosa, indicating an aversive affect and dysfunctional regulation strategies dimension. Factor 2 was labelled “**harmful substance use**” and included alcohol and drug dependency. Factor 3 was labelled “**fear-related (social) perceptions**”. It included social anxiety disorder, specific phobia, and psychosis, capturing symptoms with a social-perceptual or fear-based content.

**Table 3:** Factor loadings of *quartimax*-rotated principal axis factor analysis (*N* = 678 patients with chronic tinnitus) across all obtained diagnosis pointers.

		Rotated sums of squared loadings		
		1 (Eigenvalue: 3.04; 25.35% variance)	2 (Eigenvalue: 1.21; 10.11% variance)	3 (Eigenvalue: 1.09; 9.04% variance)
Measure				
ISR	majdep	**0.72**		
	agora	**0.76**		
	ocd	**0.77**		
	hypoch	**0.59**		
	anorex	**0.46**		
SCID-I	socP	**0.37**		**0.38**
	specP	**0.46**		**0.49**
	bulim	**0.55**		**0.35**
	alcd		**0.79**	
	drugd		**0.63**	
	mania_bid			
	psycho			**0.67**

*Notes*. Boldened coefficients indicate main factor loading. ISR = ICD-10 Symptom Rating; SCID = Structured Clinical Interview for Mental Disorders; majdep = Major Depressive Disorder; agora = Agoraphobia with or without Panic Disorder; ocd = Obsessive-Compulsive Disorder; hypoch = Health Anxiety; anorex = Anorexia Nervosa; socP = Social Anxiety Disorder; specP = Specific Phobia; bulim = Bulimia Nervosa; alcd = Alcohol Dependency; drugd = Drug Dependency; and psycho = Psychosis.

To assess whether this factor structure was consistent across patient subgroups, we computed additional factor analyses separately for patients with and without HL. Despite some variation in cross-loadings, the core factor structure was retained across groups. It emerged almost identically in patients without HL, whereas patients with HL showed a slightly different configuration: in this subgroup, fear-related (social) perceptions appeared as Factor 2, with bulimia additionally loading on this factor—possibly further highlighting a role of socially mediated emotional distress. Factor 3 indexed harmful substance use and also included manic symptoms, potentially reflecting shared psychological dynamics with substance use, such as impulsivity or loss of control. Psychosis cross-loaded on Factors 1 and 2, pointing to its complex positioning at the intersection of emotional distress and perceptual dysregulation

Examining associations between the factor scores and TRD or HL status respectively, regression analyses revealed that Factor 1 significantly predicted TRD (*β*_*Factor1*_ = 0.57; *t* = 16.94; *p* <.001, 95% CI [21.42, 27.15]). For Factor 3, a non-significant trend emerged (*β*_*Factor3*_ = 0.07; *t* = 1.92; *p* =.06, 95% CI [−0.04, 3.31]). By contrast, Factor 2 (but not Factors 1 or 3) predicted HL status such that higher scores were associated with *reduced odds* of HL (*B* = –0.36, *SE* = 0.11, *Wald* = 10.54, *p* =.001, *Exp(B)* = 0.70, 95% CI [0.56, 0.87]). However, this effect appeared to be influenced by age: univariate analyses revealed a robust linear decline in Factor 2 scores with age (*B* = –0.018, *SE* = 0.002, *F*(1, 676) = 66.37, *p* <. 001, *η*^*2*^ =. 089), indicating substantially higher substance-related scores in younger adults compared to older participants.

## Discussion

The present study examined the prevalence and covariation of psychopathology and HL in a clinical population of *N* = 678 patients with chronic tinnitus. We first compared self-reported diagnostic prevalence in patients with vs. without HL and tested whether HL status was associated with differences in TRD severity. Next, we examined which diagnosis pointers predicted TRD and whether these associations differed by HL status. We then explored the covariation structure among the diagnosis pointers using factor analytic techniques and identified three emergent transdiagnostic factors. Finally, we tested whether these factors were associated with TRD and HL status, respectively.

### High proportion of chronic tinnitus patients without hearing loss

As outlined in the Introduction, HL is the most well-established risk factor for the initial onset of tinnitus^2^. In our sample, however, more than half of patients (52.1%) exhibited no identifiable HL across the main speech frequencies. This proportion aligns with prior estimates which reported that approximately 40% of tinnitus patients presented with clinically normal audiograms^51,68^. Although we acknowledge that standard audiometry may fail to detect hidden cochlear pathology^69^, dead regions^70,71^, or high-frequency HL^72–74^, the substantial number of cases lacking a clear otological explanation nonetheless challenges the often implicit equation of chronic tinnitus with HL—particularly in the context of distressing tinnitus presentations.

### Extensive psychological vulnerability, especially in patients without hearing loss

From this perspective, chronic tinnitus may be best understood as an expression of psychological vulnerability, instantiated in auditory experience: A persistent auditory percept whose chronicity and impact largely reflect broader patterns of emotional vulnerability and meaning-making; not discrete psychiatric ‘disorders’ or peripheral pathology “per se”. Rather than dismissing audiological factors, this view emphasizes the interplay between auditory input and individual psychological context, in which past or present distress can shape how a stimulus (the tinnitus symptom) is processed, experienced and maintained over time.

Our findings support this interpretation: Consistent with previous research^75^, the current sample showed substantial psychological vulnerability, with 96% of patients endorsing at least one diagnosis pointer (median and mode = 5). Depression was the most frequently reported problem^42^, followed by various anxiety disorders and maladaptive coping strategies, including substance use.

Although all patients reported considerable psychological burden, those with HL showed higher rates of agoraphobia (with or without panic disorder), obsessive-compulsive disorder, bulimia nervosa, and drug dependency, but lower rates of anorexia nervosa, compared to those without HL. The observation that psychological comorbidity was equally—and in some cases more—pronounced among individuals without identifiable auditory deficits challenges models that attribute chronic tinnitus primarily to peripheral pathology. Instead, it supports the view of chronic tinnitus as a marker of heightened psychological vulnerability, reflected in distinct profiles of emotional distress which are *not* contingent on sensory impairment. Individuals without HL may represent a particularly vulnerable subgroup in whom the tinnitus percept emerges independently and is subsequently imbued with distressing interpretations. In this group, psychological vulnerability may serve as both initiating and maintaining factor, whereas in those with HL, it may primarily perpetuate and exacerbate an initially audiologically grounded symptom. Overall, these findings support the conceptualization of chronic tinnitus not as a uniform audiological disorder but as a psychologically mediated *process* in which chronicity and clinical relevance are largely determined by individual differences in psychological functioning^16,47,76,77^.

### Diagnosis pointers and prediction of tinnitus-related distress

Overall, the sample experienced ‘moderate’ levels of TRD. After controlling for key demographic and clinical variables, individuals with HL reported slightly higher levels of distress than those without HL. Given that psychological vulnerability was elevated in both subgroups, we do not interpret this pattern as evidence that HL “causes” distress, but that a comparable psychological vulnerability unfolds in different auditory contexts. In patients with HL, reduced peripheral input and increased listening effort may render the tinnitus signal more salient in everyday situations, providing more frequent opportunities for threat-focused appraisal and worry. In patients without HL, the same vulnerability may contribute more directly to the emergence and maintenance of tinnitus despite otherwise intact hearing. In both groups, it is therefore the interaction between underlying vulnerability and the auditory context—rather than HL alone—that appears central to explaining differences in TRD.

Regression analyses revealed that a substantial proportion (39%) of TRD-variance could be explained by the given diagnosis pointers. Across the full sample, major depressive disorder, agoraphobia (with or without panic disorder), health anxiety, anorexia nervosa, and psychosis emerged as significant predictors of TRD—most aligning with the internalizing spectrum as defined in the HiTOP. Notably, agoraphobia showed a stronger predictive effect in individuals with HL. While prior research has documented elevated anxiety rates among people with HL^78^, the underlying psychological mechanisms remain underexplored. The present findings may reflect sensory degradation associated with HL, which heightens situational vulnerability, spatial exposure, crowd sensitivity, and sensory overload. Together with an internal focus of attention, these factors likely interact with a dispositional tendency to interpret benign physiological sensations in a catastrophic manner—core features of agoraphobic anxiety^79,80^. HL may amplify this attentional bias, reinforcing maladaptive interoceptive monitoring and fear-based interpretations. Crucially, this shared vulnerability may also account for the distress observed in chronic tinnitus, wherein a neutral auditory percept (the tinnitus symptom) becomes fear-laden and persistently distressing. This framework helps explain why the associations between agoraphobia and TRD emerge regardless of HL status, but are especially pronounced among chronic tinnitus patients with HL.

### Dimensions of psychopathology

In our sample, factor analytic techniques identified three dimensions of psychopathology—internalizing psychopathology (Factor 1), harmful substance use (Factor 2), and fear-related (social) perceptions (Factor 3)—which together accounted for 57.09% of the variance in the diagnosis pointers’ covariation. These dimensions mapped meaningfully onto the HiTOP model, with Factor 1 corresponding to the *distress* subfactor of the *internalizing spectrum*, Factor 2 to the *harmful substance use* subfactor of the *externalizing spectrum*, and Factor 3 to the *fear* subfactor of the *internalizing spectrum*^81^.

Factor 1 primarily reflected conditions associated with low mood and anxiety. This finding supports both the crucial importance (and shared variance) of these constructs for chronic tinnitus^39,42^ and supports dimensional approaches that account for their frequent co-occurrence^82–84^.

Factor 2 captured harmful substance use, reflecting findings on a role of substance use in patients with chronic tinnitus—although the available evidence to this regard is mixed^85^ – partly owed to the heterogeneity of psychological reasons for substance use^86–88^.

Factor 3 captured fear-related (social) perceptions, including self-reported diagnoses of social anxiety disorder, specific phobia, and psychosis. These conditions share features of heightened fear, social threat sensitivity, and altered perceptual or interpretive processes, aligning conceptually with the *fear* subfactor of the *internalizing spectrum* in the HiTOP model. Although psychosis is traditionally grouped under the thought disorder spectrum in psychiatric nosologies, self-reported psychosis in non-psychotic clinical samples often reflects psychosis-like experiences at the milder end of a continuum, rather than the severe and enduring presentations that current systems classify as psychotic disorders. This is consistent with evidence that unusual beliefs and perceptual experiences are relatively common in the general population^89–92^ and can co-occur with anxiety and social evaluative concerns. In our view, Factor 3 therefore indexes a fear- and perception-focused vulnerability pattern, potentially modulated by social-evaluative or perceptual processes^93^, rather than indicating a distinct “psychotic” subgroup of tinnitus patients. More broadly, the findings underscore the importance of a transdiagnostic perspective that embeds chronic tinnitus perception in patients’ wider psychological makeup and guides biological research toward correlates of vulnerability dimensions, rather than discrete diagnostic labels.

### Prediction of tinnitus-related distress and hearing loss status

The identified dimensions of psychopathology further predicted TRD or HL, respectively. Internalizing psychopathology (and, at trend level, fear-related [social] perceptions) significantly predicted TRD but not HL status. By contrast, harmful substance use was associated with HL status, but not TRD. Because descriptive analyses indicated a clear age-related decline in harmful substance use, with younger individuals (especially those aged 22–35) reporting the highest levels of substance use, this latter relationship likely reflects an age confound, whereby younger participants engage in more substance use but report less HL due to age-related preservation of hearing^94^. This pattern supports viewing distressing chronic tinnitus primarily as a centrally mediated phenomenon that resides squarely on the internalizing spectrum of psychopathology, rather than as a consequence of peripheral auditory damage.

## Limitations

The present study has several important limitations. First, psychiatric diagnoses—already operational constructs with known validity limitations—were approximated here using further simplified, screening-based definitions, rather than fully structured clinical interviews. A similar limitation concerns our use of screening-based diagnosis pointers rather than dimensional syndrome scales. This choice prioritized clinical interpretability and comparability between the ISR and SCID-I screening data, yet necessarily involved some loss of information which may have attenuated subtle between-group differences. Future work should extend our approach by modelling dimensional symptom measures directly, ideally at the item level, within a HiTOP-consistent framework.

Second, findings from the exploratory factor analysis should be interpreted tentatively due to the methodological limitations inherent in this approach. Tetrachoric correlation coefficients are to be understood as association indices of binary variables treated ‘as if’ they were ordinal. Whilst this method is the methodological approach of choice^95,96^, some researchers have raised validity concerns regarding the resulting factor structures^97,98^. Moreover, factor analytic outcomes are model-dependent, shaped by variable selection and theoretical decisions made throughout the modeling process. Future studies should aim to replicate, validate, and further interpret the factor structure observed here.

Moreover, in the current study, the conceptual mapping between empirically derived factors and HiTOP spectra was based on the diagnostic *content* of each factor rather than fine-grained, symptom-level data. Future research should strengthen these insights by directly assessing dimensional symptom constructs.

Finally, cross-sectional data provide only preliminary associations and cannot capture the temporal dynamics of symptom development. Although we interpret the broad and transdiagnostic symptom patterns as more consistent with pre-existing psychological vulnerability, it is also conceivable that the emergence of a persistent tinnitus percept contributes to subsequent increases in distress-related symptoms, thereby creating a mutually reinforcing cycle between tinnitus and internalizing psychopathology. While more well-controlled longitudinal studies are needed to disentangle potential bidirectional processes, existing findings suggest that pre-existing vulnerability-shaped stimulus processing tendencies predict tinnitus chronification^7,77^—which is consistent with the conceptual framework adopted in the present study. Future research should incorporate comprehensive assessments of internalizing symptoms and evaluate the effects of transdiagnostic interventions targeting psychological vulnerabilities in patients with chronic tinnitus^99^.

## Conclusion

Taken together, the findings support a view in which peripheral auditory factors may contribute to the onset and salience of tinnitus, whereas its persistence and associated distress are more strongly tied to broader patterns of psychological vulnerability. Internalizing-type symptom load covaried robustly with TRD in patients both with and without HL, suggesting that similar vulnerability patterns may be relevant across patients with and without HL. Within the constraints of our screening-based, cross-sectional design, these results indicate that chronic tinnitus is unlikely to be psychologically neutral and occurs in context of broader internalizing and somatoform difficulties.

The results, which will require longitudinal work to further evaluate, suggest at least two subpopulations: In one, audiologically driven tinnitus may be more likely to become chronic when it encounters pre-existing psychological vulnerability; in others, similar predispositions may play a stronger role in shaping the emergence, appraisal, and maintenance of tinnitus in the relative absence of measurable HL. In both cases, it is the pattern of psychological vulnerability—rather than HL per se—that appears to underlie distressing symptom persistence. This perspective aligns with current clinical guidelines, which identify psychological therapies as the only universally recommended treatment—precisely because they target such transdiagnostic processes^100^. Clinically, our findings support a shift from circumscribed, symptom-focused psychiatric models toward contextualized, formulation-driven care^14,101,102^, which situates tinnitus within patients’ broader transdiagnostic patterns of emotional functioning and appraisal, and attends to how these patterns influence the psychological impact of the symptom^103^.

## Data Availability

As per Charité Universitatsmedizin Berlin’s ethics committee, unfortunately we cannot make the data public without restrictions because we did not obtain patients’ consent to do so at the time. Nevertheless, interested researchers can contact the directorate of the Tinnitus Center (birgit.mazurek@charite.de) or the Charité’s Open Data and Research Data Management Officer Dr. Evgeny Bobrov (evgeny.bobrov@charite.de) with specific data access requests.

## Footnotes

1 Originally, the dataset comprised 696 patients. Nine patients did not provide HL-, and 9 patients did not provide diagnostic data – and were thus excluded from the sample.
