# Correction: Transdiagnostic dimensions of psychopathology in chronic tinnitus patients with and without hearing loss

**DOI:** 10.1038/s41598-026-42649-y

**Published:** 2026-03-03

**Authors:** Benjamin Boecking, Kurt Steinmetzger, Petra Brueggemann, Matthias Rose, Birgit Mazurek

**Affiliations:** 1 Tinnitus Center, Charité – Universitatsmedizin Berlin, Charitéplatz 1, 10117 Berlin, Germany; 2 Department of Psychosomatic Medicine, Charité – Universitatsmedizin Berlin, Berlin, Germany

Correction to: *Scientific Reports* 10.1038/s41598-025-32526-5, published online 16 January 2026

The original version of this Article contained an error in the names of authors Benjamin Boecking, Kurt Steinmetzger, Petra Brueggemann, Matthias Rose & Birgit Mazurek, which were incorrectly given as Boecking Benjamin, Steinmetzger Kurt, Brueggemann Petra, Rose Matthias & Mazurek Birgit.

The original Article has been corrected.

